# Chirps and Sentiments regarding PMS and PMDD: where does X stand?

**DOI:** 10.1192/j.eurpsy.2025.2386

**Published:** 2025-08-26

**Authors:** C. Noureddine, G. Alvim de Paula, A. Sterchele

**Affiliations:** 1 Psychiatry, Icahn School of Medicine at Mount Sinai, New York, United States

## Abstract

**Introduction:**

The emergence of social media platforms like X has created a unique space for mental health discussions. This study aims to analyze the language and themes used in social media discussions to appreciate sentiments about PMS and PMDD by looking at a sample of the most popular tweets on platform X.

**Objectives:**

We hypothesize that this content can provide insight into public perceptions and guide educational campaigns.

**Methods:**

An advanced Twitter/X search for “PMS” and “PMDD” was conducted, filtering for English content. The top 100 tweets for each search term were explored through two different sentiment analysis tools which include Dr. Daniel Soper’s Sentiment Analyzer Tool and Text2Data Application Programming Interface (API) Natural Language Processing (NLP) Analysis. Tweets were also analyzed using a word cloud generator to identify the most frequently used terms. Connecting words were eliminated from the final output.

**Results:**

Negative sentiment was more prevalent than positive among the tweets for PMDD, with an overall sentiment analysis of an average of -24.3 per the Daniel Soper Sentiment Analyzer tool, suggesting a negative and serious tone. The most frequently appearing terms in these tweets were “month” (mentioned 17 times), “bad” (16), “love” (15), “feel” (14), “MAFS” (14), “support (14). Per Text2Data’s API NLP analysis, the top 150 words had a negative sentiment of -0.59 with a magnitude of 1.69. Negative sentiment was more prevalent than positive among the tweets for PMS, with an overall sentiment analysis of an average of -15.2 per the Daniel Soper Sentiment Analyzer tool, suggesting a somewhat negative and serious tone. The most frequently appearing terms in these tweets were “new” (11), “price” (10), “oxford” (9), “feel” (14), “people” (9), “want” (9). Per Text2Data’s API NLP analysis, the top 150 words had neutral sentiment of +0.15 with a magnitude of 1.59.

**Conclusions:**

This study emphasizes a less negative sentiment associated with PMS compared to PMDD. It also highlights how more emotionally charged terms were used among tweets discussing PMDD compared to PMS. This may reflect public perception of the two conditions. Additionally, social media can be a way to gauge public interest and perception of medical topics.

**Disclosure of Interest:**

None Declared

